# Development and Clinical Evaluation of a CRISPR-Based Diagnostic for Rapid Group B *Streptococcus* Screening

**DOI:** 10.3201/eid2709.200091

**Published:** 2021-09

**Authors:** Lingxiao Jiang, Weiqi Zeng, Wanting Wu, Yingying Deng, Fusheng He, Wenli Liang, Mingyao Huang, Hong Huang, Yongjun Li, Xiaorui Wang, Hang Su, Shilei Pan, Teng Xu

**Affiliations:** Zhujiang Hospital, Southern Medical University, Guangzhou, China (L. Jiang, Y. Deng, W. Liang, M. Huang);; Vision Medicals Center for Medical Research, Shenzhen, China (W. Zeng, W. Wu, H. Huang, Y. Li, X. Wang, H. Su, T. Xu);; Key Laboratory of Animal Gene Editing and Animal Cloning in Yunnan Province and College of Veterinary Medicine, Yunnan Agricultural University, Kunming, China (W. Zeng, T. Xu);; Zhujiang Hospital, Southern Medical University, Guangzhou (S. Pan)

**Keywords:** group B Streptococcus, CRISPR, rapid diagnostic, screening, bacteria, streptococci

## Abstract

Vertical transmission of group B *Streptococcus* (GBS) is among the leading causes of neonatal illness and death. Colonization with GBS usually is screened weeks before delivery during pregnancy, on the basis of which preventive measures, such as antibiotic prophylaxis, were taken. However, the accuracy of such an antenatal screening strategy has been questionable because of the intermittent nature of GBS carriage. We developed a simple-to-use, rapid, CRISPR-based assay for GBS detection. We conducted studies in a prospective cohort of 412 pregnant women and a retrospective validation cohort to evaluate its diagnostic performance. We demonstrated that CRISPR-GBS is highly sensitive and offered shorter turnaround times and lower instrument demands than PCR-based assays. This novel GBS test exhibited an overall improved diagnostic performance over culture and PCR-based assays and represents a novel diagnostic for potential rapid, point-of-care GBS screening.

Group B *Streptococcus* (GBS) is a common commensal bacteria of vaginal flora with reported carriage rates of 4%–40% (*1*–*3*). Vertical transmission of (GBS) through fetal aspiration of infected amniotic fluid or during birth canal passage has been considered one of the most important causes of neonatal illness and death (*3*,*4*). GBS colonization during pregnancy has been a leading cause of severe neonatal infectious diseases, including sepsis, pneumonia, and meningitis (*5*,*6*). Early onset neonatal infections can be prevented in most cases by providing intrapartum antibiotic prophylaxis to the colonized mother (*7*). However, GBS carriages are often intermittent, and the rate of GBS colonization varies during pregnancy (*1*,*8*). On the other hand, use of antibiotic prophylaxis solely relying on risk assessment leads to unnecessary treatment in many women. Therefore, determination of colonization at the time of delivery is crucial for the prevention of neonatal infection (*9*).

Culture-based methods remain the most commonly used screening practice and the standard for GBS detection; however, because of technical limitations, including turnaround time, pregnant women are usually screened for GBS at 35–37 weeks of gestation (*6*). As many studies have pointed out, the predictive value of GBS decreases as the interval time increases between screening and delivery (*10*,*11*). These studies underlie the needs for a more rapid and sensitive diagnostic for intrapartum GBS screening.

CRISPR/Cas has been widely used as a programmable tool for gene editing and other in vivo applications since 2013 (*12*–*14*). However, recently, the collateral, promiscuous cleavage activities of a unique group of Cas enzymes were discovered and harnessed for in vitro nucleic acid detection (*15*–*17*).

To address the unmet clinical needs for GBS screening, we developed CRISPR-GBS, a novel CRISPR/Cas13-based in vitro diagnostic assay, and conducted a prospective cohort study and a validation study in >400 clinical cases to evaluate its diagnostic performance among different technology platforms, including culture and PCR-based methods. Our findings demonstrate that CRISPR-GBS is rapid and easy-to-use, having a low instrument requirement and a level of sensitivity that surpasses PCR-based assays.

## Materials and Methods

### Study Participants and Sample Collection

A total of 426 pregnant women were prospectively admitted into Zhujiang Hospital (Guangzhou, China) for antenatal care during March 7–November 22, 2019. We excluded 14 from this cohort study because of insufficient samples for testing, incomplete clinical or experimental data, or invalid test results attributable to internal control failures. We included the remaining 412 samples in the prospective cohort study, in which direct culture, direct clinically validated PCR, and CRISPR-GBS tests were performed for each patient.

We conducted the validation cohort retrospectively, where we performed direct culture and CRISPR-GBS. For the purpose of validation, we included for enrichment culture 31 samples consisting of about one third each of dual-positive, dual-negative and discordant samples, according to the results of direct culture and CRISPR-GBS.

We collected vaginal–rectal swab specimens from the enrolled patients. Sample collection was reviewed and approved by the Zhujiang Hospital Ethics Committee Review Board. Informed consents were signed by patients or their surrogates. 

### Cas13a Protein

After codon optimization, we synthesized the open reading frame (ORF) of Cas13a and cloned it by using Gene Services (Genscript Biotech, https://www.genscript.com). The Cas13a ORF expression vector was transfected into *Escherichia coli* BL21. We first grew transfected cells at 37°C and then incubated them with isopropyl β-d-1-thiogalactopyranoside at 16°C. We purified proteins from lysed bacteria by using the Ni-NTA protocol (*18*) and stored aliquots of purified protein at −80°C.

### Strains and Human DNA

We purchased the *S. agalactiae* (group B *Streptococcus*) strain from the American Type Culture Collection (ATCC13813). *S. pneumoniae*, *S. pyogenes*, *S. mitis*, *Enterococcus faecalis*, *Acinetobacter baumannii*, and *Pseudomonas aeruginosa* strains were donated by China’s National Institutes for Food and Drug Control. We purchase another 2 species of bacteria, *E. coli* and *Staphylococcus aureus*, from China’s General Microbiological Culture Collection Center. We purchased pure human DNA from Solarbio (http://www.solarbio.net), which we eluted in nuclease-free water.

### Oligos and gRNA

Primer with an appended T7 promoter used in the recombinase polymerase amplification (RPA) for *atoB* amplification were forward primer 5′-TAAT ACGA CTCA CTAT AGGG AATT GAAT GGAA TGAA CCAT TTGC AGCG AT-3′ and reverse primer 5′-AATA ATTC CTGA GCAG GCAT AAGG GTGT C-3′. We used sgRNA for Cas13 (5′-GGGG AUUU AGAC UACC CCAA AAAC GAAG GGGA CUAA AACU CUCU CUUC AGGA UAAU AAUG AUUA AAU-3′) and ssRNA probe (5′-6-FAM-UUUUUC-BHQ1) for CRISPR detection after RPA amplification. Primer used in the nested PCR amplification for *atoB* amplification for round 1 were forward primer 5′-ACGG AAAA ACTA TTAA CAGA AACT CATA CT-3′ and reverse primer 5′-AATA ATTC CTGA GCAG GCAT AAGG GTGT C-3′ and for round 2 were forward primer 5′-CTCA TACT AAAA TATC GGAT TATG ATGC-3′ and reverse primer 5′-AGGC ATAA GGGT GTCC GTAA GC-3′.

### DNA Rapid Extraction

We eluted swabs with 1 mL of saline. We transferred 200 μL of eluate to a new sterile, nuclease-free 1.5-mL tube. After a 5-minute centrifugation at 10,000 × *g*, we resuspended the pellet in lysis buffer consisting of 0.1% sodium dodecyl sulfate and 1% NP40. We added glass microbeads and used a Crystal Industries vortex mixer (https://crystalindustries.com) to disrupt the bacterial cell walls. We then heated samples at 99°C for 10 min and centrifuged them again at 14,000 × *g*. We used 2 μL of supernatant as template for each subsequent assay for GBS detection.

### CRISPR-GBS

The CRISPR-GBS test combines an RPA step and a subsequent T7 transcription and Cas13 detection step, as described previously (*17*). In brief, we incubated reactions containing 2 μL of sample, 0.4 μM of each primer, 1 × reaction buffer, 14 mM of magnesium acetate, and the RPA enzyme mix at 37°C for 30 min. Then we added the amplification product to the CRISPR reaction mix, consisting of 33.3 nM of gRNA, 66.7 nM of Cas13, 5 mmol/L of each nucleotide triphosphate, 1 μL of T7 RNA polymerase (New England Biolabs, https://www.neb.com) and 166 nM of ssRNA reporter. We incubated the final reaction mix at 37°C and monitored it for fluorescence signal. We collected fluorescent signals by using an ABI7500 qPCR machine (ThermoFisher Scientific, https://www.thermofisher.com) for 20 min.

### Evaluation of Limit of Detection

**Figure 6:**
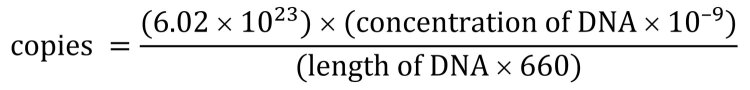
Number of genomic copies by using the formula.

For the evaluation of limit of detection by the number of genomic copies, we purified DNA of the GBS strain (ATCC13813) and determined the concentration by using Qubit (ThermoFisher Scientific). We calculated the number of genomic copies by using the formula (Figure 6).

We performed serial dilution with nuclease-free water to achieve desired concentrations. For the evaluation of limit of detection by CFU per mL, we serially diluted a reference ATCC strain with known CFU with a negative sample to the desired titer before subjecting it to DNA extraction. Although accurate conversion is challenging, our and others’ observations comparing DNA quantity and CFU counts showed that 1 CFU equaled ≈3–5 genome copies (data not shown) (*19*).

We used 2 μL of extracted DNA at each titer as templates. We performed 10 replicates at each data point. 

### Direct Culture and Enrichment Culture

We eluted each swab with 1 mL of saline. For direct culture, we inoculated 200 μL of eluate onto selective chromogenic GBS screening media (CHROMID Strepto B; bioMérieux, https://www.biomerieux-diagnostics.com) and incubated it at 37°C for 24 h aerobically. We incubated negative plates for another 24 h before the final plate reading. For enrichment culture, we first inoculated 200 μL of swab eluate into selective Todd Hewitt broth and incubated it at 37°C aerobically overnight. We then inoculated the enriched broth onto chromogenic Brilliance GBS agar (bioMérieux) by using the same experimental procedures as direct culture. We subjected all suspect colonies to Lancefield streptococcal grouping to confirm GBS.

### PCR and Nested PCR

We performed the regular PCR testing by using a validated commercial GBS PCR kit (BEC, http://www.biochainbj.com) according to the manufacturer’s instructions. We performed the nested PCR assay in 2 successive rounds of amplification. The first round amplified a larger fragment of the *atoB* gene for 35 cycles. We then subjected 2 μL of the primary PCR product to the second amplification by using a nested set of primers targeting a shorter fragment as part of the first amplicon. We then purified the amplicons from the second round and subjected them to Sanger sequencing for validation. We considered positive only those samples that both yielded PCR products after the second round of amplification and had sequences validated by Sanger.

### Statistical Analysis

We conducted comparative analysis by using Pearson χ^2^ test, Fisher exact test, or the Student *t*-test, where appropriate. We performed data analyses by using SPSS Statistics 22.0 (IBM, https://www.ibm.com). We considered p values <0.05 as statistically significant. All tests were 2-tailed unless indicated otherwise.

## Results

### Development of CRISPR-GBS

**Figure 1:**
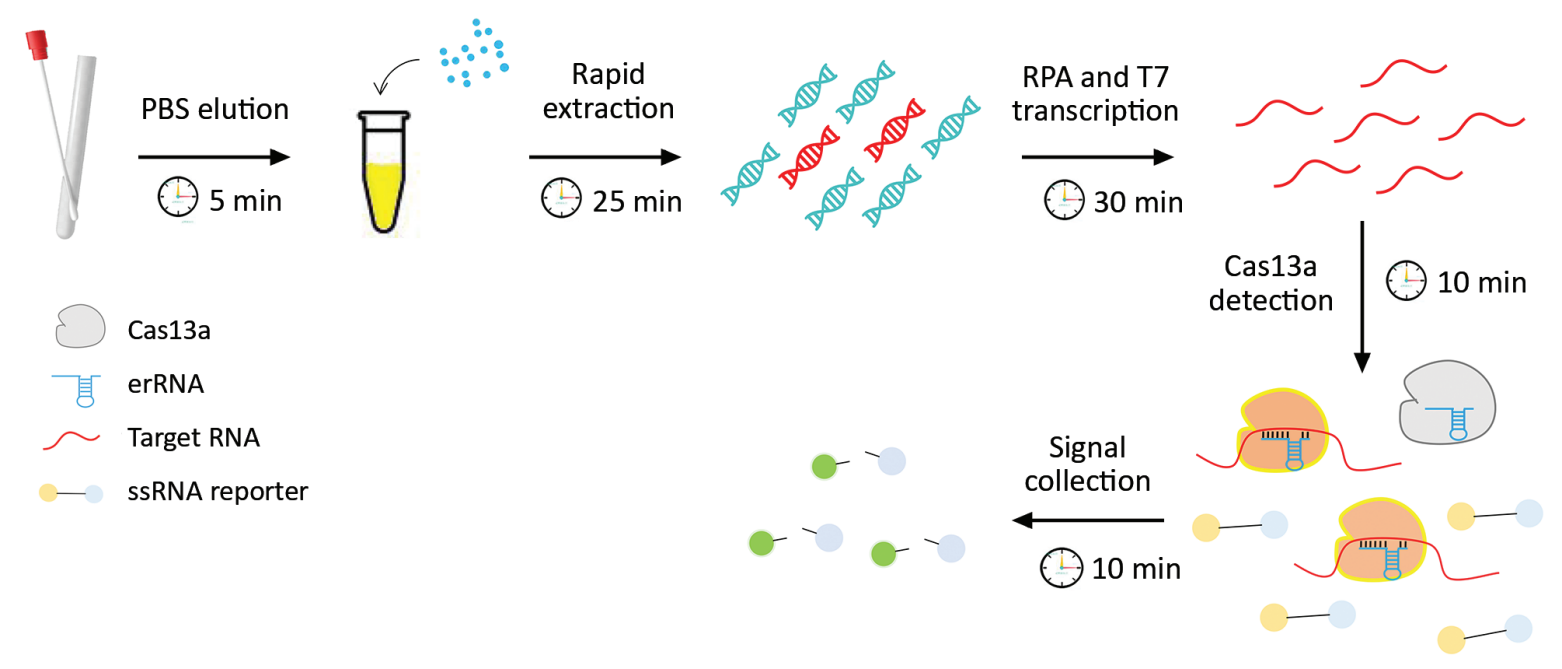
Schematic diagram of CRISPR-based diagnostic for rapid GBS screening. Swab samples are first eluted and followed by a rapid DNA extraction step where the bacterial cell walls are disrupted by a combination of chemical, physical, and heating effects. The extracted DNA is then subjected to the CRISPR/Cas reaction. The collateral nuclease activity of Cas proteins are activated upon specific binding of gRNA to the *atoB* gene. Fluorescent signal produced from cleaved probes is captured and indicates the presence of GBS. GBS, group B *Streptococcus*. gRNA, guide RNA; ssRNA, single-stranded RNA.

To address the challenges in clinical GBS screening, we aimed to develop a rapid, highly sensitive, and simple-to-use GBS assay by combining an RPA reaction with a CRISPR/Cas13 step for target detection (*17*). We established a rapid extraction method for high efficiency GBS DNA extraction by combining chemical, heat, and bead beating-based cell wall disruption, which eliminated the need for any column and organic solvents (Figure 1; Appendix Figure 1). This strategy takes advantage of both the polymerase-mediated DNA amplification and the CRISPR/Cas-mediated enzymatic signal amplification for greater sensitivity. Moreover, the rapid extraction and isothermal nature of such an assay eliminated the demand for sophisticated instruments such as thermal cyclers.

**Figure 2:**
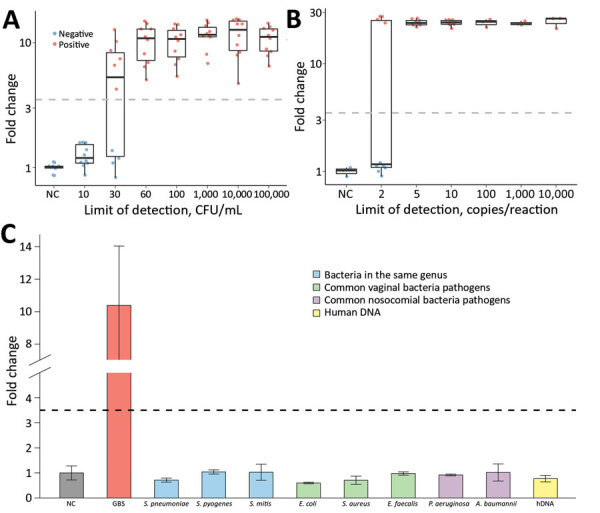
Analytical assessment of the sensitivity and specificity of CRISPR-based diagnostic for rapid GBS screening. Evaluation was performed by testing contrived negative swab samples with indicated CFUs of GBS (A), different copy numbers of GBS genomic DNA (B), and various microbes as interfering materials (C). GBS, group B *Streptococcus*. A. baumannii, *Acinetobacter baumannii*; *E. coli*, Escherichia coli*;* *E. faecalis*, *Enterococcus faecalis*; hDNA, human DNA; *P.aeruginosa*, *Pseudomonas aeruginosa*; *S. aureus, Staphylococcus aureus*; *S. mitis*, *Streptococcus mitis*; *S. pneumoniae*, *Streptococcus pneumoniae*; *S. pyogenes*, *Streptococcus pyogenes*.

We chose the thiolase (*atoB*) gene as the target region in this assay because it is highly conserved and specific for the GBS genome (*20*). We screened multiple sets of RPA primers and CRISPR gRNAs targeting different regions within *atoB* (Appendix Table 2, Figure 2). The set that showed the best overall performance of sensitivity and specificity was then used in this study for assay optimization and clinical diagnostic evaluation.

We then sought to determine the analytical sensitivity by serial dilutions of GBS with negative swabs at various counts of CFU per mL. CRISPR-GBS managed to detect samples at 30 CFU/mL in 6 of 10 runs and at 60 CFU/mL in all 10 replicates (Figure 2, panel A). We further assessed the limit of detection of CRISPR-GBS by titrations of copies per reaction. The CRISPR assay consistently detected 5 copies of GBS in 10 of 10 runs and 2 copies in 4 of 10 replicates (Figure 2, panel B). These data indicate that CRISPR-GBS could detect a low number of genome copies or ≈50 CFU/mL and is more sensitive than most of the commercially available US Food and Drug Administration–approved GBS assays, such as GeneXpert GBS (300 CFU/mL) (Cepheid, https://www.cepheid.com), BD Max GBS (1,000 CFU/mL) (BD, https://www.bd.com), Quidel Solana GBS (2.6 × 10^5^ CFU/mL) (Quidel, https://www.quidel.com), and AmpliVue GBS (1.4 × 10^6^ CFU/mL) (Quidel) (*20*–*22*).

With such a high sensitivity of CRISPR-GBS, we set out to confirm its specificity. For this purpose, we assayed DNA from humans and a panel of bacteria, including bacteria in the same genus (e.g., *S. pneumonia*, *S. pyogenes*, and *S. mitis*), microbes commonly found in vaginal swabs (e.g., *E. coli*, *Staphylococcus aureus*, and *Enterococcus faecalis*), and bacteria commonly found in nosocomial infections (e.g., *Acinetobacter baumannii* and *Pseudomonas aeruginosa*) (*23*). Of note, none of these interference samples triggered a false-positive reaction (Figure 2, panel C). Altogether, these analytical evaluations suggest that CRISPR-GBS, with its great sensitivity and specificity, is a promising molecular assay for GBS detection.

### Clinical Diagnostic Evaluation of CRISPR-GBS

After the analytical study, we further assessed the diagnostic potential of CRISPR-GBS in settings of clinical screening. A total of 426 pregnant women with a median age of 29 years (20–47 years) were enrolled in this cohort study. Sample collection was performed at 34–38 weeks of gestation. Among these patients, 14 were excluded because of invalid test results, an insufficient specimen, or both. The remaining 412 patients were tested for GBS by culture, PCR, and CRISPR-GBS on their direct swab samples. We found no significant differences between patients who were negative or positive for GBS on the basis of patient age or weeks of gestation (Appendix Table 1).

**Figure 3:**
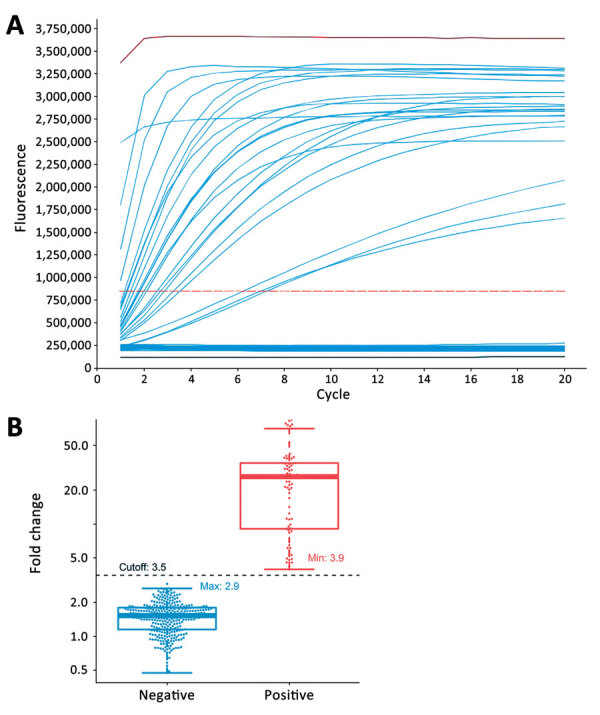
Determination of assay cutoff for CRISPR-based diagnostic for rapid GBS screening. A) Representative signal curves produced by CRISPR-GBS. A positive control (red), a negative control (black), and 85 clinical samples (blue) are shown with distinct curve patterns (take-off vs. flat). B) Fold-change values by CRISPR-GBS obtained from our prospective cohort: positive (with take-off signal curves in red) and negative (flat curves in blue). A cutoff of 3.5 was set and is indicated in black dashed line. GBS, group B *Streptococcus*. Lines from the bottom to the top of box-and-whisker plots refer to minimum, first quartile (Q1), median, third quartile (Q3), and maximum number of the dataset.

When we conducted the CRISPR-GBS assay, we included a positive control of GBS DNA and a no-template control in parallel for each run. We used a fluorescent signal from no-template control normalize the signal of other samples in the same run to calculate the corresponding fold changes. We noticed clear distinctions in signal patterns of the reactions. Specifically, the fluorescent signal curve either remained flat (e.g., the no-template control runs) or had a distinguishable takeoff from the baseline (e.g., the positive control runs) (Figure 3, panel A). To determine the cutoff value as fold-changes for the CRISPR-GBS results, we first separated all the runs into a tentatively positive group and a tentatively negative group according to these distinct patterns. We then analyzed the cutoff values. The tentatively positives had fold changes ranging from 3.9 to 90.3 (median 26.3), whereas the tentatively negatives ranged from 0.5 to 2.9 (median 1.5) (Figure 3, panel B; Appendix Figure 3). Therefore, we were able to set the cutoff value at 3.5 for complete separation of the 2 groups. Consistently, this cutoff was further confirmed by the receiver operating characteristic analysis for optimal sensitivity and specificity (data not shown).

To evaluate the diagnostic performances of different methodologies for GBS detection, we began by comparing direct culture and PCR. We found a concordance of 97.1% between these 2 traditional methods. Specifically, only 5 (1.2%) of 412 culture-positive and 7 (1.7%) of 412 PCR-positive cases were missed by the other test. When culture was used as the reference standard, PCR demonstrated a sensitivity of 90.9% (50/55 results) and specificity of 98.0% (350/357 cases).

**Figure 4:**
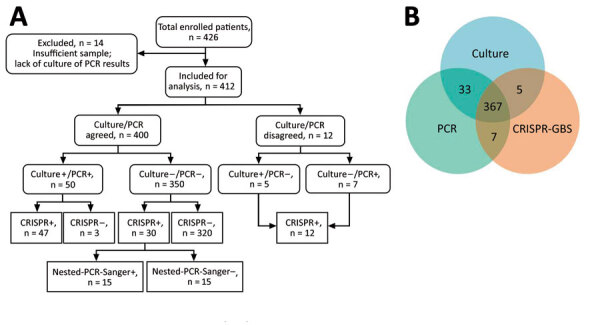
Overview and summary of the prospective cohort study assessing CRISPR-GBS. A) Study enrollment and result summary as categorized by agreements between different tests. B) Venn diagram demonstrating the overall concordance and discordance among direct culture, regular PCR, and CRISPR-GBS in the cohort. CRISPR-GBS, CRISPR-based diagnostic for rapid group B *Streptococcus* screening.

We further assessed the CRISPR-GBS test in comparison with direct culture and the PCR-based assay (Table; Figure 4). When the comparison was made separately, CRISPR-GBS was able to detect most of the positive samples by either reference method, with a sensitivity of 94.5% (52/55 cases) compared with culture and 94.7% (54/57 cases) compared with PCR. When we included only the 400 cases with concordant culture and PCR results in the analysis, CRISPR identified 94.0% (47/50) of the positive results and offered a negative predictive value of 99.1% (320/323 cases).

**Table:** Positive and negative agreement of CRISPR-based diagnostic for rapid group B *Streptococcus* screening versus different reference standards*

Assay and result	CRISPR-GBS			% (95% CI)							
				Sensitivity		Specificity		Positive predictive value		Negative predictive value	
	Positive	Negative	Total								
Direct culture												
Positive	52	3	55		94.5 (83.9–98.6)		89.6 (85.9–92.5)		58.4 (47.5–68.6)		99.1 (97.1–99.8)	
Negative	37	320	357									
Total	89	323	412									
PCR												
Positive	54	3	57		94.7 (84.5–98.6)		90.1 (86.4–92.9)		60.7 (49.7–70.7)		99.1 (97.1–99.8)	
Negative	35	320	355									
Total	89	323	412									
Direct culture and PCR												
Positive	47	3	50		94.0 (82.5–98.4)		91.4 (87.9–94.0)		61.0 (49.2–71.7)		99.0 (97.1–99.8)	
Negative	30	320	350									
Total	77	323	400									
Enriched culture												
Positive	22	0	22		100 (81.5–100.0)		100 (62.9–100.0)		100 (81.5–100.0)		100 (62.9–100.0)	
Negative	0	9	9									
Total	22	9	31									

Among the cases reported negative by culture, PCR, or both, we also found ≈10% of them to be positive by CRISPR, which included 37 of 357 culture-negative cases, 35 of 355 PCR-negative cases, and 30 of 350 dual-negative cases (i.e., by culture and PCR). These data indicate a greater sensitivity or a lower specificity of CRISPR-GBS.

We designed and conducted additional validation studies in an attempt to validate the improved sensitivity of CRISPR-GBS. We developed a nested PCR–Sanger assay targeting the *atoB* gene, in which we performed 2 successive rounds of PCR in a nested manner to achieve greater amplification sensitivity compared with regular single-round PCR reactions. We then subjected the amplicons to Sanger sequencing for further validation. With this nested PCR assay, we tested the 30 specimens that were only positive by CRISPR-GBS but negative by both direct culture and regular PCR in our cohort. We were able to confirm 15 of 30 discordant cases (Figure 4, panel A). These data supported the previous findings and again indicate higher sensitivity of CRISPR-GBS compared with direct culture or PCR.

**Figure 5:**
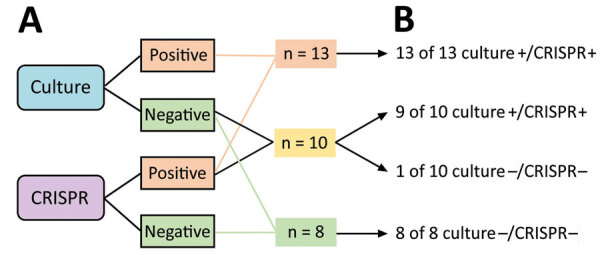
Overview of the validation study with enrichment culture for CRISPR-based diagnostic for rapid group B *Streptococcus* screening. Testing results by culture and CRISPR before (left) and after (right) broth enrichment are shown.

To further rule out the possibility of false-positive results, we set up a retrospective validation study and compared the sensitivity of CRISPR-GBS with enrichment culture, which had been shown to be more sensitive than direct culture (*5*,*24*). The validation cohort of 31 patients consisted of 13 CRISPR-positive and direct culture–positive, 10 CRISPR-positive and direct culture–negative, and 8 CRISPR-negative and direct culture–negative samples. We tested each sample by direct culture, enriched culture, and CRISPR-GBS both before and after broth enrichment. We performed enriched culture by overnight culture in selective broth, followed by inoculation onto blood agar. We found that the samples that were negative by both direct culture and CRISPR originally would remain negative even after broth enrichment. However, of the 10 cases that were positive by CRISPR but negative by direct culture, adding the broth enrichment step yielded positive results in 90% of those cases (Figure 5). These results validated the greater sensitivity of CRISPR and suggested that the testing direct swabs by CRISPR-GBS conferred comparable sensitivity as enrichment culture. In our antepartum cohort of 412 pregnant women, the prevalence of GBS carriage was the highest by CRISPR at 21.6% (89/412) and was similar by culture (13.3% [55/412]), and PCR (13.8% [57/412]).

When we compared turnaround time, we found that the CRISPR-GBS test required an average of <1.5 hours, which includes 30 minutes of rapid DNA extraction, 30 minutes for DNA amplification by RPA, and 20 minutes for Cas13 detection. This turnaround time is a considerable advantage over those for conventional culture-based (24–60 hours) and PCR-based (≈2.5 hours for a regular PCR assay and much longer for nested PCR–Sanger) methods.

## Discussion

We developed and demonstrated a CRISPR-based assay that offered short turn-around time and great sensitivity, which makes it a potential rapid, point-of-care assay for intrapartum GBS diagnosis, even in low-resource settings. Debates have occurred over approaches of preventing neonatal diseases caused by GBS infection (*25*). However, both of the 2 commonly used conventional strategies (i.e., risk-based screening or late antenatal microbiologic testing) have their own limitations (*3*,*26*). A point-of-care, rapid intrapartum GBS diagnosis at the onset of labor or membrane rupture is highly desired clinically because it would enable more accurate antibiotic prophylaxis and better antimicrobial stewardship (*5*). Successful development of such a diagnostic has been hindered by its requirement for a combination of short turn-around time, high diagnostic performance, low technical complexity, and low instrument requirement. In our study, we took advantage of the programmable CRISPR/Cas system for GBS detection. The CRISPR-GBS assay as established and demonstrated in our study takes <1.5 hours to complete, has a sensitivity comparable to enriched culture, and does not require any sophisticated instruments. These features illustrate its great potential to be an onsite, rapid diagnostic for intrapartum GBS screening. Given the low complexity of the CRISPR-GBS assay established in our study, integration of the entire testing into a compact desktop instrument for an automated sample-in-report-out assay is highly feasible.

In our prospective study, we found the prevalence of GBS in our cohort to be slightly higher than 20% by CRIPSR. Although studies have shown differential prevalence between rectal and vaginal screening, the question of whether this could be caused by a lack of assay sensitivity for detecting borderline bacterial level remains controversial (*1*,*24*,*27*). In current clinical practice, vaginal–rectal swab specimens are commonly collected for optimized GBS detection, despite reported discomfort or even pain associated with rectal swabs (*28*,*29*). Determining whether patients could be spared the discomfort of rectal specimens without compromising the results with a more sensitive assay would be worthwhile. With this sensitive and rapid CRISPR assay, further studies are also warranted to evaluate its diagnostic and clinical value as an intrapartum assay by comparing it to antepartum culture (*30*).

Apart from GBS diagnosis, obtaining the information on drug susceptibility is also of great clinical value. For instance, recent reports have showed a trend of increased erythromycin and clindamycin resistance internationally (*31*–*33*). Genotypic analysis has been proven to have great predictive value for drug resistance. Given the highly sensitive nature of this CRISPR diagnostic technology, it holds the potential to simultaneously detect genes related to drug susceptibility (*34*). An expanded CRISPR-GBS assay would be able to not only diagnose GBS colonization but also provide genetic insight into drug susceptibility for first-line antibiotics. On the basis of the proof-of-principle demonstrated in our study for direct-from-swab testing, rapid CRISPR detection of both pathogen and drug sensitivities would permit the precise approach to identification of GBS colonization and prevention of related neonatal diseases.

Because GBS is an important infection agent for multiple invasive infectious diseases such as meningitis, CRISPR-GBS could also be a promising tool for potentially much wider applications. A future multicenter study with a larger cohort would provide a more thorough evaluation of its diagnostic value, including its performance under different clinical settings. 

In summary, the CRISPR-based rapid GBS assay we established in this study exhibits great diagnostic performance for GBS colonization under analytical and clinical settings. This novel test offers improved diagnostic performance over culture- and PCR-based assays and represents a novel option for potential rapid, point-of-care GBS screening. 

Appendix Additional information about development and clinical evaluation of a CRISPR-based diagnostic for rapid group B *Streptococcus* screening.

## References

[1] Dillon HC Jr, Gray E, Pass MA, Gray BM. Anorectal and vaginal carriage of group B streptococci during pregnancy. J Infect Dis. 1982;145:794–9. 10.1093/infdis/145.6.794 7045248

[2] American Academy of Pediatrics Committee on Infectious Diseases and Committee on Fetus and Newborn. Guidelines for prevention of group B streptococcal (GBS) infection by chemoprophylaxis. Pediatrics. 1992;90:775–8. 1408555

[3] Melin P. Neonatal group B streptococcal disease: from pathogenesis to preventive strategies. Clin Microbiol Infect. 2011;17:1294–303. 10.1111/j.1469-0691.2011.03576.x 21672083

[4] Phares CR, Lynfield R, Farley MM, Mohle-Boetani J, Harrison LH, Petit S, et al.; Active Bacterial Core surveillance/Emerging Infections Program Network. Epidemiology of invasive group B streptococcal disease in the United States, 1999-2005. JAMA. 2008;299:2056–65. 10.1001/jama.299.17.2056 18460666

[5] Verani JR, McGee L, Schrag SJ; Division of Bacterial Diseases, National Center for Immunization and Respiratory Diseases, Centers for Disease Control and Prevention (CDC). Prevention of perinatal group B streptococcal disease—revised guidelines from CDC, 2010. MMWR Recomm Rep. 2010;59(RR-10):1–36. 21088663

[6] The Royal Australian and New Zealand College of Obstetricians and Gynaecologists. Maternal group B *Streptococcus* in pregnancy: screening and management (C-Obs 19). 2019 [cited 2021 Jul 19]. https://ranzcog.edu.au/RANZCOG_SITE/media/RANZCOG-MEDIA/Women%27s%20Health/Statement%20and%20guidelines/Clinical-Obstetrics/Maternal-Group-B-Streptococcus-in-pregnancy-screening-and-management-(C-Obs-19).pdf

[7] Schrag SJ, Zywicki S, Farley MM, Reingold AL, Harrison LH, Lefkowitz LB, et al. Group B streptococcal disease in the era of intrapartum antibiotic prophylaxis. N Engl J Med. 2000;342:15–20. 10.1056/NEJM200001063420103 10620644

[8] Hansen SM, Uldbjerg N, Kilian M, Sørensen UB. Dynamics of *Streptococcus agalactiae* colonization in women during and after pregnancy and in their infants. J Clin Microbiol. 2004;42:83–9. 10.1128/JCM.42.1.83-89.2004 14715736 PMC321715

[9] Valkenburg-van den Berg AW, Houtman-Roelofsen RL, Oostvogel PM, Dekker FW, Dörr PJ, Sprij AJ. Timing of group B streptococcus screening in pregnancy: a systematic review. Gynecol Obstet Invest. 2010;69:174–83. 10.1159/000265942 20016190

[10] Boyer KM, Gadzala CA, Kelly PD, Burd LI, Gotoff SP. Selective intrapartum chemoprophylaxis of neonatal group B streptococcal early-onset disease. II. Predictive value of prenatal cultures. J Infect Dis. 1983;148:802–9. 10.1093/infdis/148.5.802 6355317

[11] Yancey MK, Schuchat A, Brown LK, Ventura VL, Markenson GR. The accuracy of late antenatal screening cultures in predicting genital group B streptococcal colonization at delivery. Obstet Gynecol. 1996;88:811–5. 10.1016/0029-7844(96)00320-1 8885919

[12] Hwang WY, Fu Y, Reyon D, Maeder ML, Tsai SQ, Sander JD, et al. Efficient genome editing in zebrafish using a CRISPR-Cas system. Nat Biotechnol. 2013;31:227–9. 10.1038/nbt.2501 23360964 PMC3686313

[13] Wu Y, Liang D, Wang Y, Bai M, Tang W, Bao S, et al. Correction of a genetic disease in mouse via use of CRISPR-Cas9. Cell Stem Cell. 2013;13:659–62. 10.1016/j.stem.2013.10.016 24315440

[14] Knott GJ, Doudna JA. CRISPR-Cas guides the future of genetic engineering. Science. 2018;361:866–9. 10.1126/science.aat5011 30166482 PMC6455913

[15] Chen JS, Ma E, Harrington LB, Da Costa M, Tian X, Palefsky JM, et al. CRISPR-Cas12a target binding unleashes indiscriminate single-stranded DNase activity. Science. 2018;360:436–9. 10.1126/science.aar6245 29449511 PMC6628903

[16] Harrington LB, Burstein D, Chen JS, Paez-Espino D, Ma E, Witte IP, et al. Programmed DNA destruction by miniature CRISPR-Cas14 enzymes. Science. 2018;362:839–42. 10.1126/science.aav4294 30337455 PMC6659742

[17] Myhrvold C, Freije CA, Gootenberg JS, Abudayyeh OO, Metsky HC, Durbin AF, et al. Field-deployable viral diagnostics using CRISPR-Cas13. Science. 2018;360:444–8. 10.1126/science.aas8836 29700266 PMC6197056

[18] Crowe J, Döbeli H, Gentz R, Hochuli E, Stüber D, Henco K. 6xHis-Ni-NTA chromatography as a superior technique in recombinant protein expression/purification. Methods Mol Biol. 1994;31:371–87. 7921034 10.1385/0-89603-258-2:371

[19] Parham NJ, Picard FJ, Peytavi R, Gagnon M, Seyrig G, Gagné PA, et al. Specific magnetic bead based capture of genomic DNA from clinical samples: application to the detection of group B streptococci in vaginal/anal swabs. Clin Chem. 2007;53:1570–6. 10.1373/clinchem.2007.091389 17660271

[20] Miller SA, Deak E, Humphries R. Comparison of the AmpliVue, BD Max System, and Illumigene molecular assays for detection of group B *Streptococcus* in antenatal screening specimens. J Clin Microbiol. 2015;53:1938–41. 10.1128/JCM.00261-15 25788551 PMC4432075

[21] Park JS, Cho DH, Yang JH, Kim MY, Shin SM, Kim EC, et al. Usefulness of a rapid real-time PCR assay in prenatal screening for group B *streptococcus* colonization. Ann Lab Med. 2013;33:39–44. 10.3343/alm.2013.33.1.39 23301221 PMC3535195

[22] Berry GJ, Zhang F, Manji R, Juretschko S. Comparison of the Panther Fusion and BD MAX Group B *Streptococcus* (GBS) assays for detection of GBS in prenatal screening specimens. J Clin Microbiol. 2019;57:e01034–19. 10.1128/JCM.01034-19 31462552 PMC6812996

[23] Ke D, Ménard C, Picard FJ, Boissinot M, Ouellette M, Roy PH, et al. Development of conventional and real-time PCR assays for the rapid detection of group B streptococci. Clin Chem. 2000;46:324–31. 10.1093/clinchem/46.3.324 10702518

[24] Platt MW, McLaughlin JC, Gilson GJ, Wellhoner MF, Nims LJ. Increased recovery of group B *Streptococcus* by the inclusion of rectal culturing and enrichment. Diagn Microbiol Infect Dis. 1995;21:65–8. 10.1016/0732-8893(95)00022-3 7628194

[25] Davies HD. Preventing group B streptococcal infections: new recommendations. Can J Infect Dis. 2002;13:232–5. 10.1155/2002/352613 18159394 PMC2094877

[26] Puopolo KM, Lynfield R, Cummings JJ; COMMITTEE ON FETUS AND NEWBORN; COMMITTEE ON INFECTIOUS DISEASES. Committee on Infectious Diseases. Management of infants at risk for group B streptococcal disease. Pediatrics. 2019;144:e20191881. 10.1542/peds.2019-1881 31285392

[27] Philipson EH, Palermino DA, Robinson A. Enhanced antenatal detection of group B streptococcus colonization. Obstet Gynecol. 1995;85:437–9. 10.1016/0029-7844(94)00412-7 7862387

[28] Jamie WE, Edwards RK, Duff P. Vaginal-perianal compared with vaginal-rectal cultures for identification of group B streptococci. Obstet Gynecol. 2004;104:1058–61. 10.1097/01.AOG.0000144120.20312.ed 15516402

[29] Orafu C, Gill P, Nelson K, Hecht B, Hopkins M. Perianal versus anorectal specimens: is there a difference in Group B streptococcal detection? Obstet Gynecol. 2002;99:1036–9. 10.1097/00006250-200206000-00015 12052595

[30] Iams JD, O’Shaughnessy R. Antepartum versus intrapartum selective screening for maternal group B streptococcal colonization. Am J Obstet Gynecol. 1982;143:153–6. 10.1016/0002-9378(82)90645-7 7044127

[31] Guo Y, Deng X, Liang Y, Zhang L, Zhao GP, Zhou Y. The draft genomes and investigation of serotype distribution, antimicrobial resistance of group B *Streptococcus* strains isolated from urine in Suzhou, China. Ann Clin Microbiol Antimicrob. 2018;17:28. 10.1186/s12941-018-0280-y 29945615 PMC6020191

[32] Gao K, Guan X, Zeng L, Qian J, Zhu S, Deng Q, et al. An increasing trend of neonatal invasive multidrug-resistant group B s*treptococcus* infections in southern China, 2011-2017. Infect Drug Resist. 2018;11:2561–9. 10.2147/IDR.S178717 30573985 PMC6292236

[33] Tsai MH, Hsu JF, Lai MY, Lin LC, Chu SM, Huang HR, et al. Molecular characteristics and antimicrobial resistance of group b *Streptococcus* strains causing invasive disease in neonates and adults. Front Microbiol. 2019;10:264. 10.3389/fmicb.2019.00264 30833941 PMC6387999

[34] Campisi E, Rosini R, Ji W, Guidotti S, Rojas-López M, Geng G, et al. Genomic analysis reveals multi-drug resistance clusters in group B *Streptococcus* CC17 hypervirulent isolates causing neonatal invasive disease in southern mainland China. Front Microbiol. 2016;7:1265. 10.3389/fmicb.2016.01265 27574519 PMC4983569

