# Supplementary material for: Development and Clinical Evaluation of a CRISPR-Based Diagnostic for Rapid Group B Streptococcus Screening
Source: Emerg Infect Dis. 2021 Sep;27(9):2379–88. doi: 10.3201/eid2709.200091 (PMC8386798; doi:10.3201/eid2709.200091)
Supplement: Appendix — Additional information about development and clinical evaluation of a CRISPR-based diagnostic for rapid group B Streptococcus screening. [file 20-0091-Techapp-s1.pdf]

Article DOI: https://doi.org/10.3201/eid2709.200091

Development and Clinical Evaluation of a CRISPR-Based Diagnostic for Rapid Group B *Streptococcus* Screening

Appendix

Appendix Table 1. Basic patient characteristics

	Culture Positive Group			Culture Negative Group		
Patient characteristics	Min	Max	Median	Min	Max	Median
Age (years old)	20	38	30	21	47	29
weeks of gestation	34	37	35	34	37	35

Appendix Table 2. Information of multiple RPA Primers for screening.

No.	Forward primer sequence	Reverse primer sequence
Primers #1	TAATACGACTCACTATAGGGACGGAAAAACTATTAACAGAAACTCATACT	GTAAGCTAATGTCCCTCCAAAAATATTGA
Primers #2	TAATACGACTCACTATAGGGAATTGAATGGAATGAACCATTTGCAGCGAT	AATAATTCCTGAGCAGGCATAAGGGTGTC
Primers #3	TAATACGACTCACTATAGGGTGAACCATTTGCAGCGATTGATGCTCTATT	CATAAGGGTGTCCGTAAGCTAATGTCCCT
Primers #4	TAATACGACTCACTATAGGGCGGATTATGATGCAATTGAATGGAATGAACC	CAGGCATAAGGGTGTCCGTAAGCTAATGTC
Primers #5	TAATACGACTCACTATAGGGAACTCATACTAAAATATCGGATTATGATGC	GAGCAGGCATAAGGGTGTCCGTAAGCTAATG

Appendix Table 3. Assay Readouts

Age (yr)	Gestational (wk+day)	Sample Collection Date	Ct value by qPCR	Fold-change by CRISPR-GBS
20	36	2019.03.07	25.1	42.1
24	36+1	2019.03.07	/	2.6
28	37+6	2019.03.08	/	2.2
28	36	2019.03.11	39.8	1.4
27	36+2	2019.03.11	/	2.3
32	37+	2019.03.11	/	2.4
29	35	2019.03.11	/	2.4
26	35+5	2019.03.13	26.2	39.4
28	35	2019.03.15	27.0	40.3
27	34+2	2019.03.15	/	2.2
37	35	2019.03.15	/	2.3
30	35+4	2019.03.18	38.1	32.9
34	35	2019.03.18	/	2.3
31	36+1	2019.03.19	/	2.4
25	36	2019.03.19	/	2.5
26	34+3	2019.03.19	/	2.6
27	34+3	2019.03.19	/	2.5
29	36	2019.03.20	32.1	39.9
35	35+3	2019.03.20	/	2.0
26	35+2	2019.03.20	/	2.2
33	35+1	2019.03.20	/	2.2
34	36	2019.03.20	/	2.1
31	35	2019.03.20	/	2.5
37	35+4	2019.03.22	33.3	38.8
31	35	2019.03.25	34.0	30.9
34	34	2019.03.25	/	2.4
30	34	2019.03.25	/	2.2
29	34	2019.03.25	/	2.1
22	35+5	2019.03.25	/	2.3
28	35	2019.03.25	/	2.0
35	35	2019.03.25	/	2.1
30	35	2019.03.25	/	2.2
28	35	2019.03.28	/	2.4
33	36	2019.03.28	/	2.3
25	35	2019.03.29	31.3	30.9
33	36+1	2019.03.29	/	2.3
30	34+5	2019.03.29	/	1.8
27	36	2019.03.29	/	2.2

Age (yr)	Gestational (wk+day)	Sample Collection Date	Ct value by qPCR	Fold-change by CRISPR-GBS
27	35+5	2019.03.29	/	2.2
28	37	2019.04.02	28.2	38.2
31	37+5	2019.04.02	/	1.6
34	35+4	2019.04.02	22.3	82.6
33	35+5	2019.04.02	/	1.7
28	37	2019.04.02	/	1.2
25	36+4	2019.04.03	/	1.5
29	37+3	2019.04.03	/	1.4
29	35+5	2019.04.04	/	1.9
24	37	2019.04.04	/	1.9
30	37+1	2019.04.04	/	1.7
35	37+1	2019.04.04	/	1.8
27	35	2019.04.09	31.0	26.3
30	37+1	2019.04.09	/	1.5
28	36+2	2019.04.09	/	52.5
30	35	2019.04.11	33.5	34.5
27	37	2019.04.11	/	1.2
32	35	2019.04.11	/	1.6
24	36+3	2019.04.15	26.3	70.4
36	36+6	2019.04.15	/	1.9
39	36	2019.04.15	/	2.1
34	36+6	2019.04.15	/	1.5
25	36+4	2019.04.15	/	0.8
36	37	2019.04.15	/	1.9
31	35	2019.04.16	29.8	30.2
25	36+	2019.04.16	/	2.0
29	37+2	2019.04.16	/	1.8
26	36+3	2019.04.16	/	26.4
26	36+6	2019.04.18	/	1.8
34	37+1	2019.04.18	/	20.9
23	36	2019.04.18	/	22.2
37	36+6	2019.04.18	/	26.7
28	36+4	2019.04.18	/	1.9
28	36+1	2019.04.22	/	1.8
29	36+	2019.04.22	/	23.8
27	37	2019.04.22	24.3	90.3
26	35+4	2019.04.22	/	1.7
30	37+3	2019.04.22	/	1.6
34	37+4	2019.04.23	/	1.5

Age (yr)	Gestational (wk+day)	Sample Collection Date	Ct value by qPCR	Fold-change by CRISPR-GBS
31	36+1	2019.04.23	/	1.5
33	36+5	2019.04.23	/	1.8
26	36+	2019.04.23	/	1.7
34	35+2	2019.04.23	28.8	22.7
32	36	2019.04.25	28.7	33.1
28	37	2019.04.25	/	21.1
27	36+5	2019.04.25	/	1.4
40	36+4	2019.04.25	/	1.8
31	37+3	2019.04.25	/	2.0
23	35	2019.04.25	/	1.8
29	36+2	2019.04.25	/	1.8
29	36+2	2019.04.25	/	1.7
30	36+	2019.04.29	38.1	1.7
31	37+3	2019.04.29	/	1.6
25	36	2019.04.30	21.6	78.2
31	35+3	2019.04.30	/	1.7
47	35	2019.04.30	/	1.6
32	35+3	2019.04.30	/	1.5
36	34+4	2019.04.30	/	0.9
24	37+5	2019.05.06	/	1.7
24	36+	2019.05.06	/	1.7
32	37+6	2019.05.06	/	1.7
29	34	2019.05.06	/	1.7
26	36	2019.05.06	/	1.8
28	35	2019.05.06	/	1.8
37	36+1	2019.05.06	/	1.9
29	36+1	2019.05.06	/	21.4
35	37	2019.05.07	/	1.9
24	36+1	2019.05.07	26.5	74.0
28	36	2019.05.07	/	1.6
24	36+	2019.05.07	/	1.5
34	36	2019.05.09	/	70.5
37	37+	2019.05.09	/	1.5
25	37+	2019.05.09	/	1.5
29	34	2019.05.09	/	1.5
31	35+4	2019.05.09	/	1.6
27	35+5	2019.05.09	/	1.5
40	35+1	2019.05.10	/	1.6
32	35+2	2019.05.10	29.9	76.9

Age (yr)	Gestational (wk+day)	Sample Collection Date	Ct value by qPCR	Fold-change by CRISPR-GBS
28	34+	2019.05.13	/	1.5
35	34+4	2019.05.13	/	1.5
27	34+1	2019.05.13	/	1.5
28	36+5	2019.05.14	/	1.6
38	35+1	2019.05.14	/	1.2
38	36+3	2019.05.14	/	1.8
28	36+6	2019.05.14	30.6	75.6
29	36+	2019.05.16	32.6	32.0
29	36+4	2019.05.16	/	0.9
29	34	2019.05.16	/	0.9
32	36	2019.05.16	/	0.9
29	34	2019.05.16	/	0.9
29	34+5	2019.05.16	/	0.9
26	36+2	2019.05.17	/	1.7
24	34+4	2019.05.17	/	1.6
24	37	2019.05.20	/	0.8
33	34+2	2019.05.20	/	23.8
24	36+	2019.05.20	/	26.4
23	34+4	2019.05.20	/	2.0
31	36+	2019.05.21	31.6	34.8
26	34+	2019.05.21	/	1.9
26	36+4	2019.05.21	/	0.7
22	36	2019.05.21	/	0.5
22	36+1	2019.05.21	/	1.9
37	37+1	2019.05.21	/	1.8
25	36	2019.05.21	/	1.7
28	37+	2019.05.21	/	1.9
29	37+6	2019.05.23	/	0.6
34	37	2019.05.23	/	2.0
30	36+3	2019.05.23	/	4.8
35	35	2019.05.23	/	1.8
33	34+5	2019.05.23	/	1.8
21	36+	2019.05.23	/	12.4
31	36+4	2019.05.23	/	1.8
32	36	2019.05.23	/	1.8
23	36+1	2019.05.23	/	18.8
37	36	2019.05.24	/	26.9
32	36+2	2019.05.24	/	1.9
33	36+3	2019.05.27	/	1.7

Age (yr)	Gestational (wk+day)	Sample Collection Date	Ct value by qPCR	Fold-change by CRISPR-GBS
31	36+5	2019.05.27	/	20.2
30	36+5	2019.05.28	/	1.5
30	36+4	2019.05.28	/	17.0
27	36	2019.05.28	/	2.0
36	37+3	2019.05.28	/	1.9
28	37+2	2019.05.28	/	1.9
30	35	2019.05.28	/	1.9
33	36+5	2019.05.30	30.5	34.9
30	37+1	2019.05.30	/	1.9
36	36+	2019.05.30	/	27.0
26	37+	2019.05.30	/	1.5
35	37+4	2019.05.30	/	1.6
37	35+1	2019.05.30	/	1.8
33	37+3	2019.05.30	/	0.7
31	37+6	2019.05.30	/	1.6
29	35+	2019.06.03	/	1.8
29	36+	2019.06.04	28.8	34.3
30	36+3	2019.06.04	/	1.6
37	35	2019.06.04	/	1.8
27	36+5	2019.06.04	/	1.4
29	36+4	2019.06.04	/	1.7
35	37+2	2019.06.04	/	1.7
34	36+3	2019.06.04	/	27.4
38	36+	2019.06.04	/	1.7
30	36+	2019.06.06	33.0	34.3
27	36	2019.06.06	/	1.8
30	35+3	2019.06.06	/	1.5
29	35+3	2019.06.06	26.7	84.7
28	36+	2019.06.06	/	1.5
34	36+2	2019.06.11	30.8	37.3
32	36+2	2019.06.11	/	1.5
28	37+1	2019.06.11	/	1.5
31	35+3	2019.06.12	/	1.3
31	35	2019.06.12	/	1.4
23	35+4	2019.06.12	/	1.3
24	34+5	2019.06.12	/	1.1
28	36	2019.06.12	/	1.2
31	34	2019.06.13	/	1.3
31	34+	2019.06.13	/	1.3

Age (yr)	Gestational (wk+day)	Sample Collection Date	Ct value by qPCR	Fold-change by CRISPR-GBS
28	36+5	2019.06.13	/	1.3
26	35+6	2019.06.13	34.5	1.5
25	34+3	2019.06.13	/	1.2
35	35+3	2019.06.13	/	1.7
33	35+3	2019.06.13	39.1	4.5
33	34+5	2019.06.14	/	1.1
32	36	2019.06.14	33.9	6.5
29	35+4	2019.06.14	/	2.6
30	36+4	2019.06.14	/	1.8
30	35+2	2019.06.14	/	1.1
24	36+2	2019.06.17	/	0.9
24	36+4	2019.06.17	/	1.2
26	36+2	2019.06.17	34.8	4.2
27	35+2	2019.06.17	/	8.8
26	34+2	2019.06.17	/	1.0
31	36+	2019.06.18	/	0.8
29	34+4	2019.06.18	/	11.0
23	35+1	2019.06.18	/	0.9
32	34+2	2019.06.18	22.9	14.1
33	35+6	2019.06.19	/	0.8
29	34+3	2019.06.19	/	1.4
32	35+1	2019.06.19	/	1.1
26	36+3	2019.06.19	/	0.9
25	34	2019.06.19	/	9.3
35	36	2019.06.19	/	1.0
28	36+1	2019.06.19	/	1.0
33	35+2	2019.06.19	/	4.0
27	37	2019.06.19	/	4.9
23	35+4	2019.06.19	/	1.0
31	35	2019.06.19	/	1.2
27	35+3	2019.06.20	/	1.2
33	36+2	2019.06.20	/	6.1
36	36+3	2019.06.20	/	1.1
28	35+6	2019.06.20	/	5.3
27	35+4	2019.06.20	/	0.8
29	36	2019.06.20	/	5.2
25	35+1	2019.06.20	/	1.9
36	35+3	2019.06.20	30.5	6.1
29	34+6	2019.06.20	38.1	7.0

Age (yr)	Gestational (wk+day)	Sample Collection Date	Ct value by qPCR	Fold-change by CRISPR-GBS
25	34+4	2019.06.21	32.8	8.4
36	34+2	2019.06.21	/	1.0
27	34+3	2019.06.21	/	0.5
23	35	2019.06.21	/	0.8
34	35+6	2019.07.10	/	1.1
32	35+1	2019.07.12	/	1.0
28	34+1	2019.07.12	/	1.1
33	36+1	2019.07.12	32.4	9.1
28	36	2019.07.15	/	1.2
34	36+6	2019.07.15	/	1.1
28	36+2	2019.07.15	/	1.2
30	35+6	2019.07.16	/	0.8
25	34+5	2019.07.16	/	0.9
30	36+2	2019.07.16	/	1.0
29	35+5	2019.07.16	/	1.0
37	34+6	2019.07.17	/	20.1
35	36+6	2019.07.17	/	1.0
34	34+2	2019.07.17	/	1.2
26	34	2019.07.17	/	0.9
29	34+5	2019.07.17	37.3	4.5
22	35+6	2019.07.17	/	1.0
26	35+5	2019.07.18	/	0.7
26	34	2019.07.20	/	1.0
31	34+4	2019.07.20	/	0.5
23	35+1	2019.07.20	/	0.9
35	34+1	2019.07.20	/	0.8
31	34+6	2019.07.20	/	1.6
27	34+3	2019.07.20	/	1.6
27	34+6	2019.07.20	/	1.7
27	35	2019.07.20	/	1.8
29	36+5	2019.07.20	/	1.9
26	35+6	2019.07.22	/	1.8
26	34+6	2019.07.22	/	1.7
28	35+5	2019.07.22	/	2.0
32	36+6	2019.07.22	/	1.6
28	34+2	2019.07.23	/	1.6
30	34+4	2019.07.23	/	1.6
24	34	2019.07.23	/	1.8
28	35	2019.07.23	/	2.1

Age (yr)	Gestational (wk+day)	Sample Collection Date	Ct value by qPCR	Fold-change by CRISPR-GBS
28	35+3	2019.07.23	/	1.9
25	35+6	2019.07.24	/	1.7
32	34+3	2019.07.24	/	1.6
34	34+1	2019.07.24	/	1.5
30	35+5	2019.07.24	/	1.8
37	34+	2019.07.24	/	1.6
27	34+1	2019.07.25	/	19.6
32	34+6	2019.07.26	/	1.7
26	34	2019.07.26	/	1.9
28	34+5	2019.07.29	/	2.1
30	35+4	2019.07.29	/	1.7
34	34+6	2019.07.29	/	1.7
35	34+4	2019.07.29	30.3	28.2
30	34+3	2019.07.30	/	1.7
28	34	2019.07.30	/	1.4
27	35	2019.07.30	/	1.3
30	34+5	2019.07.30	/	1.7
32	34+1	2019.07.30	/	1.0
38	34+2	2019.07.30	/	1.0
38	34+1	2019.07.30	38.6	27.8
40	36+2	2019.07.30	/	1.7
33	35+3	2019.07.30	/	1.1
35	34+4	2019.07.30	/	1.4
24	35+5	2019.07.31	/	0.9
25	34+2	2019.07.31	/	1.1
29	35	2019.07.31	36.9	8.4
31	34+2	2019.07.31	/	1.1
34	34+4	2019.07.31	/	0.8
30	35	2019.07.31	/	1.0
28	34+6	2019.07.31	/	0.8
30	34	2019.08.01	/	2.5
32	35+3	2019.08.02	/	1.1
32	34	2019.08.02	33.5	29.7
37	34+1	2019.08.02	/	1.0
23	37	2019.08.21	28.0	24.6
27	35+1	2019.08.21	/	1.1
28	35+4	2019.08.21	/	1.2
34	35+	2019.08.21	/	25.8
25	35+4	2019.08.22	/	1.4

Age (yr)	Gestational (wk+day)	Sample Collection Date	Ct value by qPCR	Fold-change by CRISPR-GBS
29	35+6	2019.08.23	/	1.2
34	36+3	2019.08.23	28.1	38.2
26	35+3	2019.08.26	/	1.1
26	34	2019.08.27	/	1.1
27	35+1	2019.08.28	/	1.2
28	35+6	2019.08.28	/	1.3
22	35+1	2019.08.30	32.6	27.8
30	35+2	2019.09.02	/	1.3
35	35+1	2019.09.02	/	1.5
24	34	2019.09.02	/	1.8
28	34	2019.09.02	/	1.6
35	35+1	2019.09.02	/	2.1
29	35+6	2019.09.02	/	1.8
32	34+2	2019.09.02	/	2.5
24	35+1	2019.09.02	/	1.5
23	34+2	2019.09.02	/	1.4
29	34+1	2019.09.02	/	1.4
28	35	2019.09.03	/	2.3
27	34+5	2019.09.03	/	1.4
28	36+4	2019.09.03	/	0.6
31	34+4	2019.09.03	/	1.6
31	34+3	2019.09.03	/	1.5
29	34+3	2019.09.03	/	1.3
27	36+6	2019.09.03	/	1.4
29	34	2019.09.03	/	1.3
33	34+5	2019.09.04	/	1.5
31	35+2	2019.09.04	/	1.6
27	35	2019.09.04	/	2.0
30	34+2	2019.09.04	/	1.4
30	34+4	2019.09.04	/	1.8
29	35+4	2019.09.04	/	1.7
36	35+	2019.09.04	/	1.4
27	35+3	2019.09.04	/	30.2
26	35+3	2019.09.04	/	1.6
31	34	2019.09.05	/	1.5
31	34+2	2019.09.05	/	1.4
30	35	2019.09.05	/	1.4
23	35	2019.09.05	/	1.6
27	35+2	2019.09.05	/	1.4

Age (yr)	Gestational (wk+day)	Sample Collection Date	Ct value by qPCR	Fold-change by CRISPR-GBS
33	34+1	2019.09.06	37.5	27.8
29	34+4	2019.09.06	/	1.4
30	34+3	2019.09.06	/	1.6
27	34+2	2019.09.06	/	1.4
32	35+1	2019.09.06	/	1.6
31	34	2019.09.09	/	1.5
26	34+1	2019.09.09	/	1.5
29	34	2019.09.09	/	1.4
27	34+2	2019.09.09	/	1.8
28	35+2	2019.09.09	/	1.4
24	35+1	2019.09.09	/	1.8
25	36+1	2019.09.09	/	1.7
29	34+2	2019.09.10	/	1.6
26	34+1	2019.09.10	/	1.6
29	35+5	2019.09.11	/	1.6
24	34+1	2019.09.11	/	1.8
32	35+5	2019.09.11	/	2.0
29	34+1	2019.09.11	/	1.5
30	34+	2019.09.11	/	1.3
29	35+5	2019.09.12	/	1.5
30	37+	2019.09.17	/	1.5
35	37+2	2019.09.18	33.9	11.1
33	37+1	2019.09.19	/	2.9
25	37+6	2019.09.20	/	1.0
34	37+3	2019.09.23	37.3	6.5
27	37+	2019.09.24	35.4	9.1
33	37+1	2019.09.25	36.8	9.5
27	37+4	2019.09.26	30.0	11.1
28	37+5	2019.10.08	/	2.5
28	37+	2019.10.10	/	2.9
32	36+3	2019.10.14	33.1	9.9
23	37+5	2019.10.16	/	0.9
25	37+2	2019.10.17	/	1.2
36	36+6	2019.10.21	/	1.3
26	37+1	2019.10.22	/	1.3
32	35+3	2019.10.23	/	0.9
36	34+4	2019.10.23	/	1.0
28	36+4	2019.10.24	38.8	4.2
29	36+4	2019.10.24	/	0.9

Age (yr)	Gestational (wk+day)	Sample Collection Date	Ct value by qPCR	Fold-change by CRISPR-GBS
38	37+3	2019.10.25	/	1.6
29	34	2019.10.25	/	4.6
32	36	2019.10.25	/	0.9
29	34	2019.10.25	/	0.6
34	37+	2019.10.28	/	2.7
29	34+5	2019.10.28	/	1.0
31	35+3	2019.10.28	/	5.8
31	35	2019.10.31	/	1.0
23	35+4	2019.11.04	/	1.5
24	34+5	2019.11.04	/	1.1
28	36	2019.11.07	/	1.2
31	34	2019.11.07	/	0.8
31	34+	2019.11.07	/	0.8
28	36+5	2019.11.07	/	4.9
26	35+6	2019.11.12	/	0.9
25	34+3	2019.11.12	/	5.5
35	35+3	2019.11.12	/	1.2
33	35+3	2019.11.12	32.8	8.6
33	34+5	2019.11.14	/	1.1
32	36	2019.11.14	33.9	7.0
29	35+4	2019.11.14	/	1.0
30	36+4	2019.11.21	/	0.8
30	35+2	2019.11.21	/	0.7

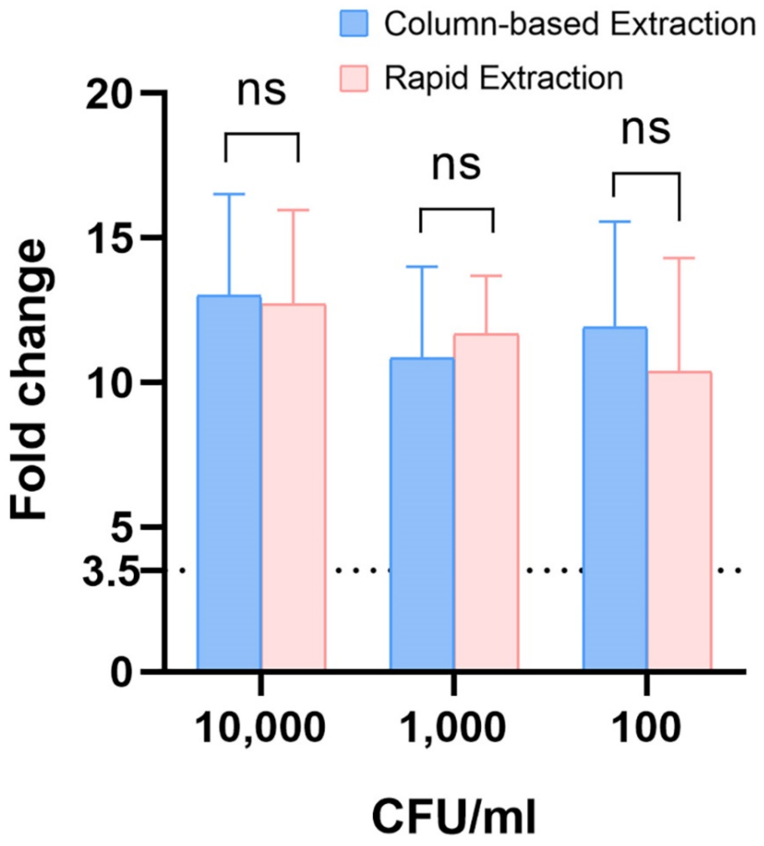

Appendix Figure 1. Comparison between column-based extraction and optimized rapid extraction. GBS samples at different CFU/ml were extracted in parallel and tested by the CRISPR assay for evaluation. N.S., not significant by the Student's t-test.

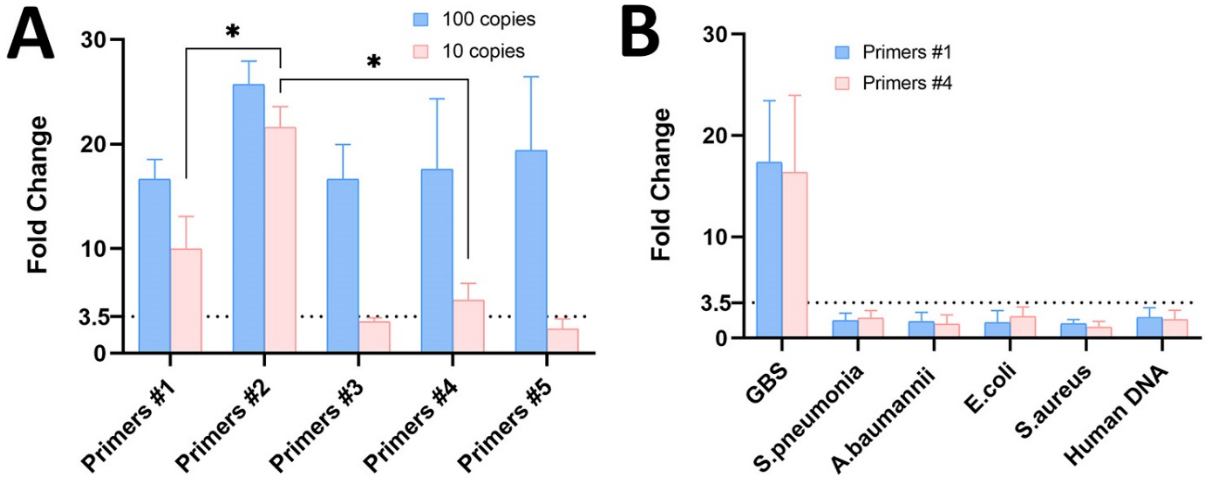

Appendix Figure 2. Pilot study for primer screening. (A) Five different pairs of primers targeting atoB genes were screened at 10 and 100 copies of GBS genomes to compare for sensitivity and signal production indicated by fold changes in flourescence. (B) Specificity testing for Primer #1 and #4 with various microorganisms as interefering materials. *, $p < 0.05$, student's t-test.

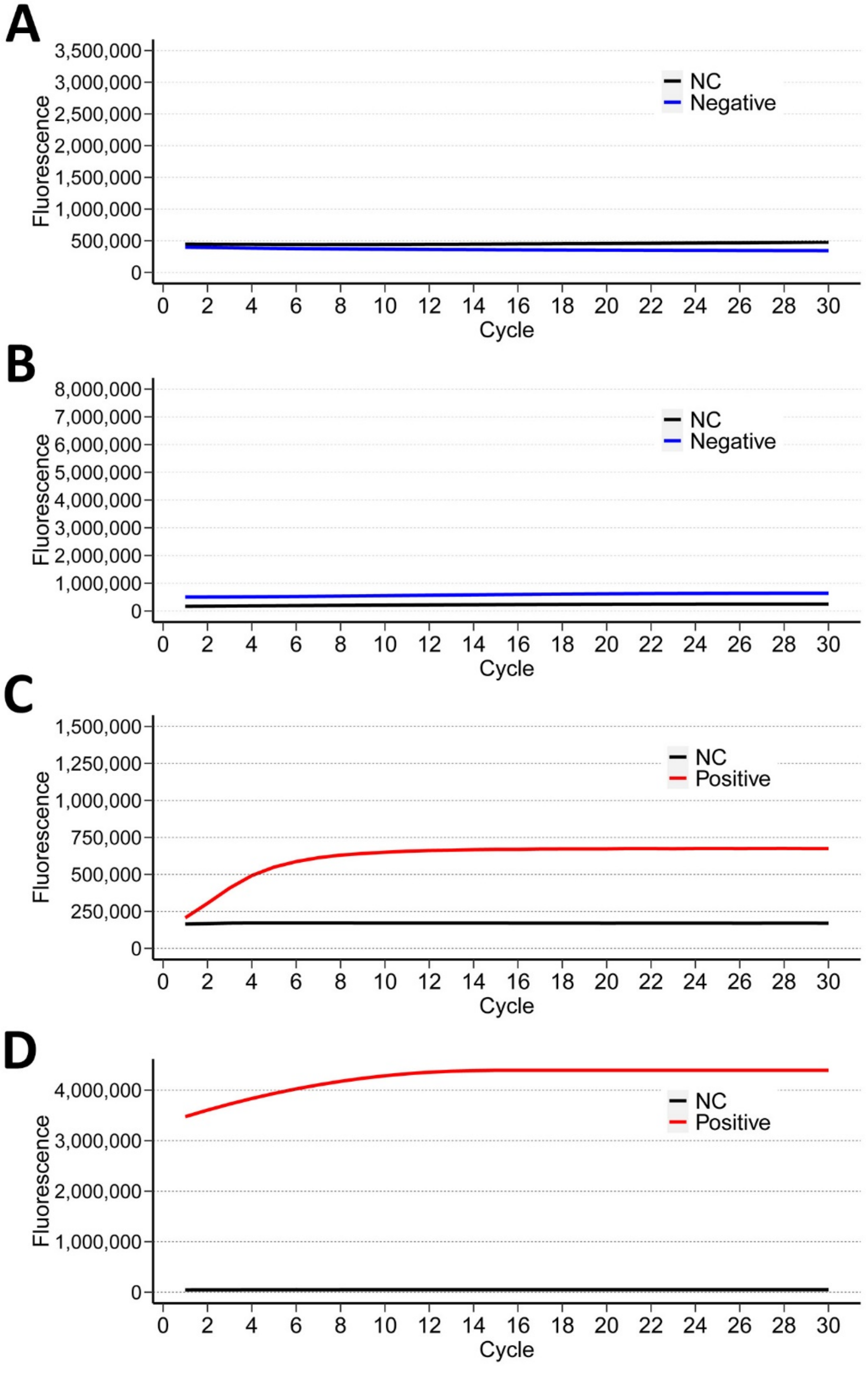

Appendix Figure 3. Representative negative and positive signal curves produced by CRISPR-GBS. (A, B) Flat, negative curves with minimal (A) and maximal (B) fold changes; (C, D) Take-off, positive curves with minimal (C) and maximal (D) fold changes. Fold-change values were 0.5, 2.9, 4.0, 90.3 for panel A, B, C and D, respectively.
